# Supplementary material for: Bergmann’s Rule under Rocks: Testing the Influence of Latitude and Temperature on a Chiton from Mexican Marine Ecoregions
Source: Biology (Basel). 2023 May 24;12(6):766. doi: 10.3390/biology12060766 (PMC10294927; doi:10.3390/biology12060766)
Supplement: Supplementary file 1 [file biology-12-00766-s001.zip › biology-2375941-supplementary.pdf]

## Supplementary Material

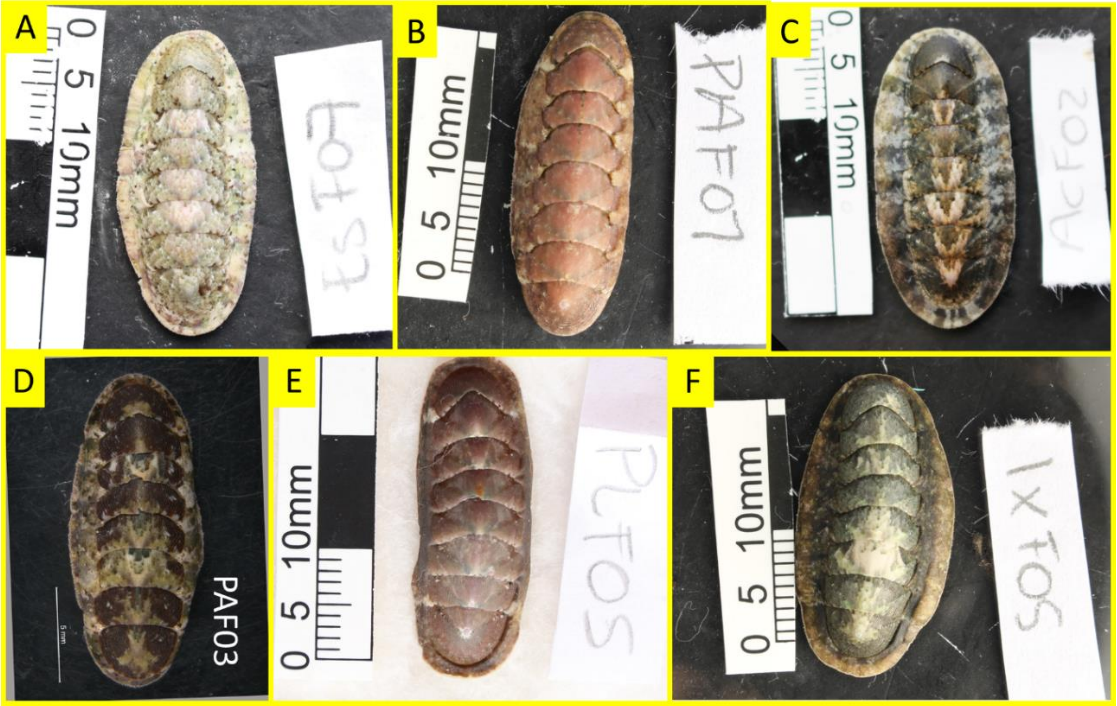


**Figure S1**. A-C. Selected individuals. D-F. Discarded individuals (D. valves without pustules; E. Crooked individual, and valves without pustules; F. Crooked individual.

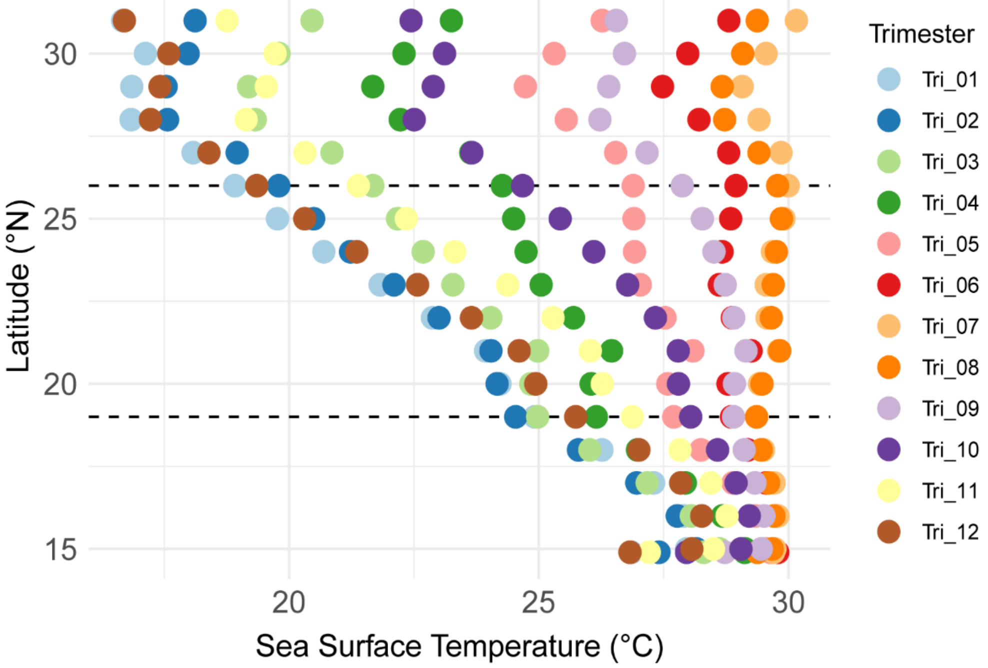


**Figure S2.** Sea Surface Temperature per trimester along the latitude, dotted lines separate the marine ecoregions.

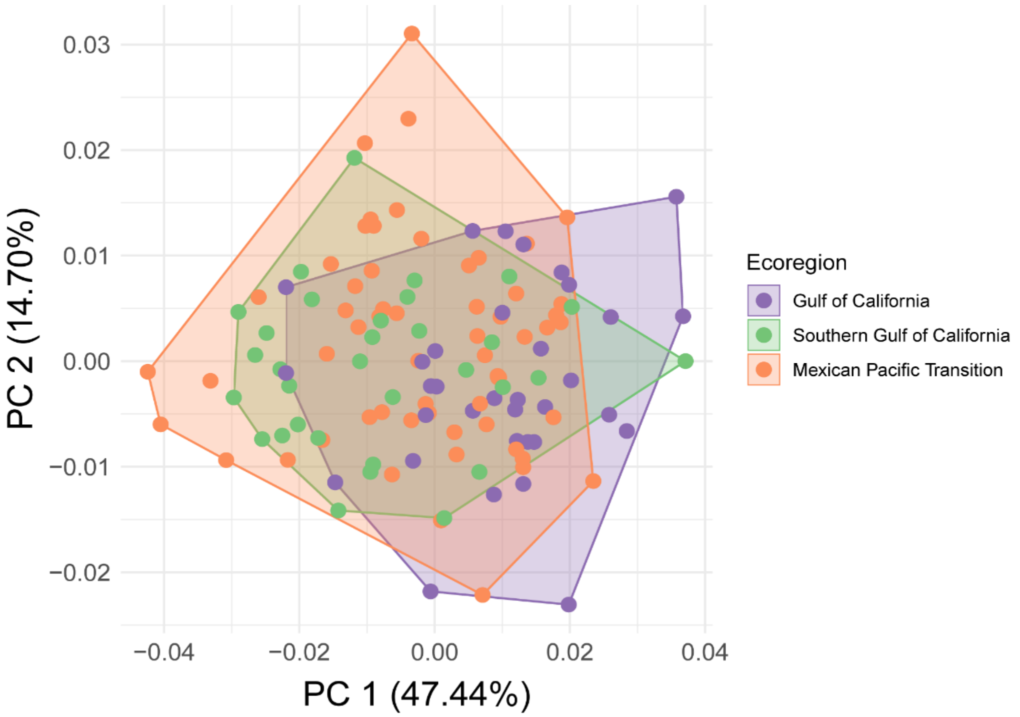


**Figure S3**. Principal Component Analysis. Polygons represent the different marine ecoregions.

**Table S1.** Sampling site information. Depth is considered during low tidal. Sites with few individuals were matched with closer sites (latitudes or coast similar) displayed by /.

| Marine ecoregion | ID | Localities | Latitude (° N) | Depth (m) | Number of individuals |
|---|---|---|---|---|---|
| Gulf of California | Pc | Puertecitos | 30.33979 | 1 | 10 |
| | PL | Sonora<br>Puerto Lobos | 30.264409 | 0 | 4 |
| | BA/KN | Bahía de los Ángeles/<br>Kino Nuevo | 29.031949/<br>28.872907 | 1.5/0.2 | 1/2 |
| | Ch | Punta Chivato | 27.069705 | 1 | 13 |
| | Jn/Sg | Baja Sur<br>(Juncalito/Sargento) | 25.843097/<br>24.34138 | 1/0.3 | 2/2 |
| Southern Gulf of California | Gy | Guaymas | 27.935749 | 7 | 9 |
| | IV | Mazatlán | 23.233749<br>23.21 | 0.20 | 13 |
| | PM | Punta Mita | 20.784561 | 1 | 10 |
| Mexican Pacific Transition | Cc | Isla Cocinas | 19.546819 | 3 | 7 |
| | IZ | Ixtapa/<br>Zihuatanejo | 17.676791/<br>17.621519 | 0.5/1 | 5/7<br>12 |
| | Ac | Acapulco | 16.844586 | 2.5 | 12 |
| | Es | Puerto Escondido | 15.857662 | 4 | 15 |
| | Hu | Huatulco | 15.760918 | 4 | 13 |

Table S2. Coordinates of satellite data of Surface Sea Temperature per latitude used on ERDDAP

| Latitude | °N | °W |
|---|---|---|
| 31 | 30.925 - 32.025 | 245.025 - 247.025 |
| 30 | 29.925 - 31.025 | 245.125 - 247.325 |
| 29 | 28.925 - 30.025 | 245.523 - 248.025 |
| 28 | 27.925 - 29.025 | 246.325 - 249.025 |
| 27 | 26.925 - 28.025 | 247.125 - 250.225 |
| 26 | 25.925 - 27.025 | 247.925 - 250.925 |
| 25 | 24.925 - 26.025 | 248.625 - 252.025 |
| 24 | 23.925 - 25.025 | 249.225 - 253.025 |
| 23 | 22.925 - 24.025 | 250.025 - 254.025 |
| 22 | 21.925 - 23.025 | 252.025 - 254.425 |
| 21 | 20.925 - 22.025 | 252.025 - 254.825 |
| 20 | 19.925 - 21.025 | 252.525 - 254.725 |
| 19 | 18.925 - 20.025 | 253.525 - 256.025 |
| 18 | 17.925 - 19.025 | 255.025 - 257.525 |
| 17 | 16.925 - 18.025 | 257.025 - 260.025 |
| 16 | 15.925 - 17.025 | 259.025 - 262.225 |
| 15 | 15.325 - 15.975 | 261.725 - 263.525 |
